# Extended Repetitive Transcranial Magnetic Stimulation Therapy for Post-stroke Depression in a Patient With a Pre-frontal Cortical Lesion: A Case Study

**DOI:** 10.3389/fneur.2022.869248

**Published:** 2022-07-14

**Authors:** Brenton Hordacre, Anson Chau, Lynton Graetz, Susan Hillier

**Affiliations:** ^1^Innovation, Implementation and Clinical Translation in Health (IIMPACT in Health), Allied Health and Human Performance, University of South Australia, Adelaide, SA, Australia; ^2^Medical Imaging, Medical Radiation Science, Allied Health and Human Performance, University of South Australia, Adelaide, SA, Australia; ^3^Alliance for Research in Exercise, Nutrition and Activity (ARENA), Allied Health and Human Performance, University of South Australia, Adelaide, SA, Australia; ^4^Department of Audiology, College of Nursing and Medicine, Flinders University, Adelaide, SA, Australia; ^5^Lifespan Human Neurophysiology, Adelaide Medical School, The University of Adelaide, Adelaide, SA, Australia; ^6^Hopwood Centre of Neurobiology, Lifelong Health Theme, South Australian Health and Medical Research Institute, Adelaide, SA, Australia

**Keywords:** depression, stroke, repetitive transcranial magnetic stimulation, maintenance therapy, treatment

## Abstract

Approximately one-third of stroke survivors experience post-stroke depression. Repetitive transcranial magnetic stimulation (rTMS) of the prefrontal cortex has shown promise as a treatment for depression with few side effects and high tolerability. However, previous post-stroke depression trials have not considered the effect of lesion location, the persistence of clinical improvements, nor the value of ongoing maintenance treatments. These questions are important to determine the therapeutic value of rTMS as a treatment for post-stroke depression. We report a unique case study of a 71-year-old male who had experienced a left hemispheric ischemic stroke 4 years prior. The patient was screened with the Beck Depression Inventory and Patient Health Questionnaire and found to be experiencing moderate levels of depression. Ten daily sessions of left dorsolateral pre-frontal cortex rTMS were applied over a two-week period. A clinically meaningful reduction in depression was achieved. Approximately 10 weeks following rTMS treatment, improvements in depression were attenuating. Weekly maintenance rTMS was delivered to the left dorsolateral pre-frontal cortex for 10 sessions. At the conclusion of maintenance rTMS, clinical assessments indicated depressive symptoms had reduced to a minimal to nil level. Clinically meaningful improvements in depression were maintained at 3 months after rTMS treatment had ceased. These findings provide novel insight to suggest rTMS may reduce depressive symptoms in stroke survivors with a lesion at the site of stimulation. Ongoing maintenance treatments might prove beneficial to enhance persistence of clinical improvements.

## Introduction

Stroke is a global leading cause of adult disability (1). Survivors are often left with permanent disability that affects quality of life (2). There are ~80 million stroke survivors worldwide, with about one third thought to experience post-stroke depression (3, 4). Those that experience depression often have poor recovery, longer hospital stays, reduced activities of daily living, reduced self-efficacy and increased mortality (3, 5–8). Indeed, post-stroke depression not only impacts mental health but also recovery. Therefore, treatments for post-stroke depression have value in promoting recovery and require consideration for improved clinical outcomes.

One treatment that might prove beneficial to manage post-stroke depression is repetitive transcranial magnetic stimulation (rTMS). Briefly, rTMS is a non-invasive method to activate synapses repeatedly in the underlying cortex. It appears to initiate early stages of synaptic plasticity *via* mechanisms that resemble long-term potentiation and long-term depression (9, 10). Ability to manipulate cortical plasticity could hold great promise as a therapeutic modality for neurological conditions that are associated with altered brain activity. In people who experience depression there is some evidence of reduced regional cerebral blood flow and neural activity of the left dorsolateral prefrontal cortex (DLPFC) that normalizes with recovery from depression (11, 12). These findings have informed many trials that have used rTMS to target the DLPFC as a treatment for depression (13, 14), with medium effect sizes and response rates of 41.5 – 56.4% in real world clinical settings (15–18). However, there remains considerable risk for relapse. Data are variable within the literature, with Dannon et al. (19) reporting a 20% relapse rate at 6-months, while Cohen et al. (20) found over 50% had relapsed at 4-months. Maintenance rTMS provides further treatment beyond the acute burst of therapy and appears that it may help prevent depressive relapse following treatment. Typically provided as a single session weekly or bi-weekly, maintenance rTMS can substantially delay or reduce occurrence of relapses (21, 22). Given the high tolerability profile and low risk of side effects, rTMS holds great promise as a treatment for depression.

Few studies have evaluated rTMS in post-stroke depression. An early trial evaluated 10 rTMS treatments in 20 depressed stroke survivors and found a significant reduction in depression 1 week after treatment (23). Similarly, in 24 chronic stroke survivors, rTMS was found to significantly improve depression, with improvements maintained at 1-month (24). More recently, 10 rTMS treatment sessions significantly improved depression following treatment and at 1-month follow-up, with clinical gains associated with greater DLPFC functional connectivity prior to treatment (25). While this limited work provides some indication that rTMS may prove beneficial as a treatment for post-stroke depression, there remain several unanswered questions. Namely, given that rTMS appears to target the DLPFC, and both cortical integrity and functional connectivity of neural targets appears to influence rTMS responses (25, 26), it is unclear whether patients with left frontal lesions would benefit from this treatment. It may be that a lesion at the site of stimulation impairs the ability of rTMS to modify neural activity, thereby reducing symptoms of depression. Furthermore, none of the previous trials investigating rTMS for post-stroke depression have evaluated persistence of improvement in depression, nor whether maintenance treatments are effective in prolonging clinical improvements. These are important questions that directly influence whether rTMS has merit as a therapy for depression after stroke and how it may be best applied in a clinical setting. Here we report a unique case study of a chronic stroke patient with a lesion of the left pre-frontal cortex who received an acute rTMS treatment course followed by a maintenance program with long-term clinical outcomes.

## Case Report

A 71-year-old male participated in a research study that delivered 2 weeks of rTMS to the left DLPFC as a treatment for post-stroke depression. The study protocol was approved by the University of South Australia Human Research Ethics Committee (200697, approval date 7/2/2018). The patient had a history of left hemispheric ischemic stroke 4 years prior, with depression onset reported within 12 months of stroke. He stated that he did not believe he was depressed prior to the stroke. At time of testing, his daily medication schedule included 50 mg Atenolol, 20 mg Rivaroxaban, 20 mg Paroxetine, 10 mg Atorvastatin, and 80 mg Ezetimibe. Medications were stable for 6 months prior to study enrolment and remained stable throughout. On presentation, the patient had notable non-fluent aphasia that appeared more pronounced in the afternoon, or with fatigue. There was minimal upper-limb impairment, scoring 65/66 for the Fugl-Meyer assessment, and a high level of upper-limb function, scoring 57/57 for the Action Research Arm Test. He was an independent community ambulator, requiring no gait aids and was rated a 5 on the Functional Ambulation Category, and had high self-efficacy, scoring 113 on the Stroke Self-Efficacy Questionnaire (range 0–130).

T1-weighted and fluid-attenuated inversion recovery (FLAIR) magnetic resonance imaging (MRI) sequences were obtained prior to rTMS treatments with a Siemens 3T MAGNETOM Skyra scanner (Siemens, Erlangen, Germany) with a 64-channel head coil. The T1 MPRAGE protocol was 1 × 1 × 1 mm voxels, repetition time = 2,300 ms, echo time = 2.98 ms, and flip angle = 9°. FLAIR images were obtained as 1 × 0.5 × 0.5 mm voxels, repetition time = 5,000 and echo time = 393 m. Image processing was performed in FSL (FMRIB Software Library, Oxford, UK). T1 images were linearly co-registered to FLAIR images and lesion masks were manually traced by an experienced investigator and lesion volume determined. Structural images were non-linearly transformed to standard space and lesion location confirmed using the Harvard-Oxford Cortical Structural Atlas within FSL.

**Figure 1:**
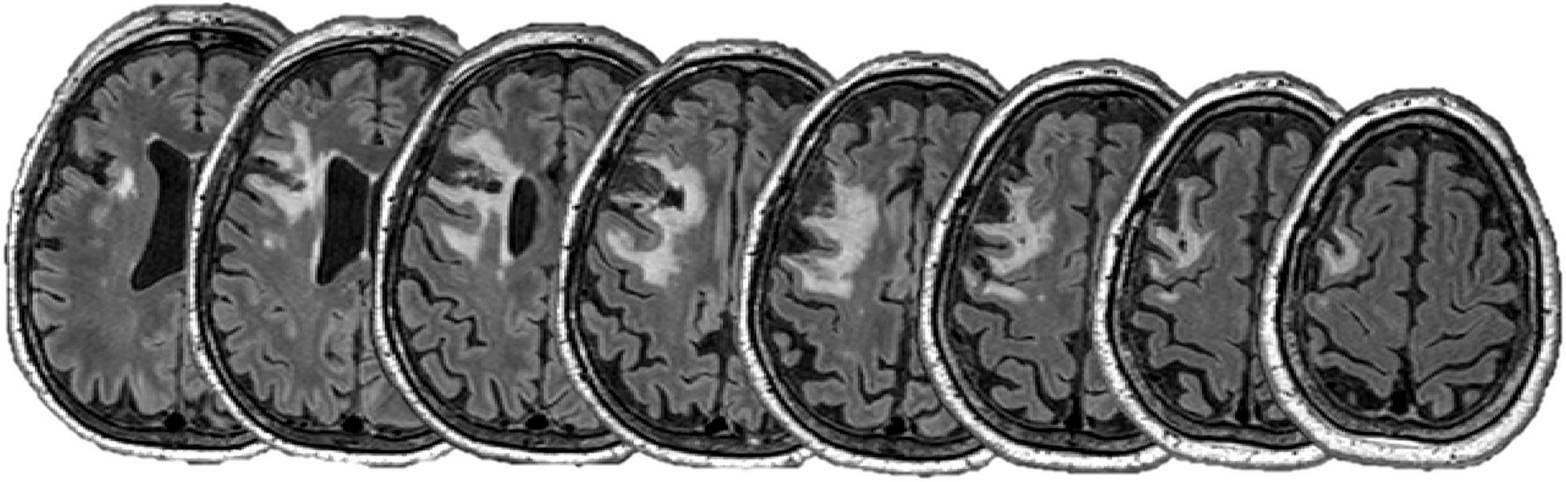
FLAIR images showing the lesion. Images are spaced by 5 mm.

The lesion was predominantly located in the left middle frontal gyrus with extension into the inferior frontal gyrus and precentral gyrus. Anatomically, the DLPFC is thought to lie within the middle frontal gyrus, within Brodmann's area 9 and 46 (27). Therefore, it appeared likely that the lesion overlapped to some extent the left DLPFC. Lesion volume was 43.0 cm^3^ and did not overlap with the corticospinal tract, suggestive of intact descending motor pathways (see Figure 1).

The patient was screened for brain stimulation and deemed safe (28), then participated in a two-week (10 session) daily treatment course of high frequency rTMS to the left DLPFC. Simulation was delivered using a Neuro-MS/D rTMS device (Neurosoft Ltd. Ivanova, Russia) that was connected to an oil cooled figure 8 coil (wing diameter 70 mm) and delivered biphasic TMS pulses. Initially, resting motor threshold was determined by an automated algorithm within the Neuro-MS software (Neurosoft Ltd. Ivanova, Russia) that identified the minimum stimulus intensity required to evoke a motor evoked potential in the relaxed first dorsal interosseous with a peak-to-peak amplitude larger than 50 μV in at least 5 out of 10 trials (pulse frequency 0.2 Hz, surface EMG 22 × 34 mm, FIAB, Florence, Italy). Resting motor threshold was determined as 31% of maximal stimulator output. All subsequent treatment sessions were delivered to the left DLPFC, located using the Beam method to identify F3 based on the 10–20 system (29), at 110% of resting motor threshold (34% maximal stimulator output). For each treatment, 3,000 pulses were delivered at 10 Hz (4 s on and 26 s off; total duration 37.5 min). We have previously found this protocol to be safe, with few side effects, when delivered to people with stroke of similar age (25, 30).

Therapeutic response to left DLPFC rTMS was assessed with the Beck Depression Inventory (BDI) and Patient Health Questionnaire 9 item scale (PHQ-9). Briefly, the BDI contains 21 items scored from 0 (symptom absent) to 3 (severe symptom), with total scores ranging from 0 to 63 (31). Scores are categorized as minimal depression (0–13), mild depression (14–19), moderate depression (20–28) and severe depression (29–63). The BDI is valid and reliable as an assessment of depression severity (32), with the minimal clinically important difference of a 17.5% improvement (33). The PHQ-9 explores nine items that are scored on a 4-point scale, ranging from 0 (no symptom) to 3 (symptom nearly every day). Scores range from 0 to 27, with 0–4 indicating no depressive symptoms, 5–9 mild symptoms, 10–14 moderate symptoms, 15–19 moderate/severe symptoms and 20–27 severe symptoms. The PHQ-9 is a valid and reliable assessment of depression (34, 35), with a 5-point reduction considered a clinically meaningful improvement (36).

**Figure 2:**
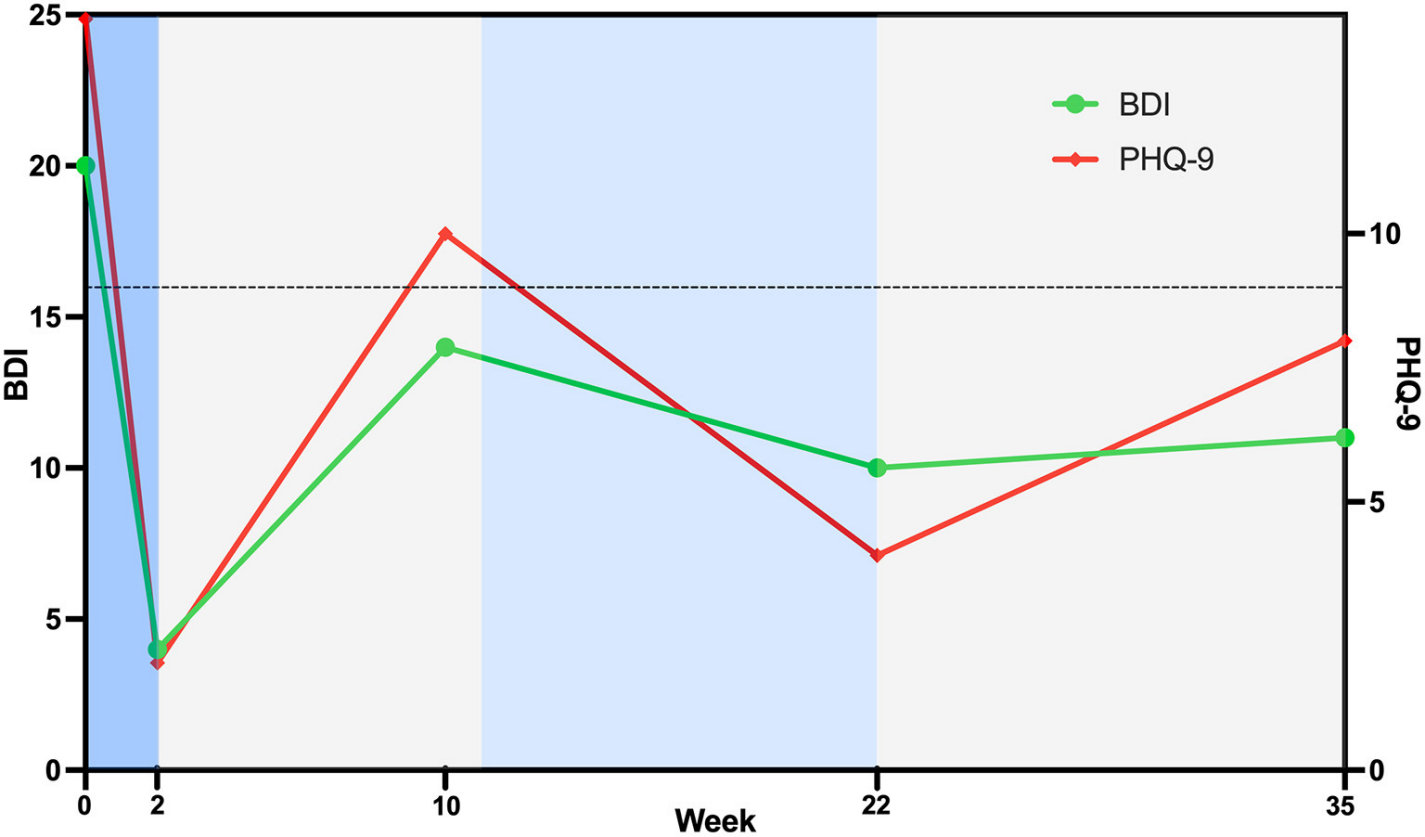
Timeline of treatment and clinical outcomes. High-frequency rTMS treatment to the left dorsolateral prefrontal cortex was delivered during the blue phases, with daily sessions from week 0–2 (darker blue) and weekly sessions from week 11–22 (lighter blue). No rTMS treatment was provided between these times. Threshold for a clinically meaningful improvement from baseline for both the Beck Depression Inventory and Patient Health Questionnaire is shown with a horizontal dotted line. Data points below the dotted line indicate a clinically meaningful improvement in depression.

Clinical outcomes and timeline are shown in Figure 2. At baseline, the outcome measures indicated that the patient was experiencing a moderate level of depression on both the BDI (score 20) and PHQ-9 (score 14). Despite this, the patient reported higher levels of self-efficacy on the Stroke Self-Efficacy Questionnaire (score 113), possibly reflecting greater confidence in functional performance due to limited motor impairments. Following 10 daily sessions of left DLPFC rTMS treatment, the patient achieved clinically meaningful reductions in depression, with both the BDI (score 4) and PHQ-9 (score 2) indicating minimal to nil symptoms. At the completion of the two-week rTMS treatment period, the patient completed an adverse events questionnaire that was adapted from Brunoni et al. (37). There were no major adverse events, with the patient only reporting mild hearing problems (reported as an increased sensation of tinnitus) and mild difficulty concentrating. Both symptoms were transient and did not bother the patient.

Approximately 10 weeks after the completion of the two-week daily rTMS treatment phase, the patient contacted the research team and identified that improvements in depression were dissipating. At this point, re-assessment of BDI (score 14) and PHQ-9 (score 10) identified a slight increase in depressive symptoms, indicative of mild-moderate levels of depression. Given previous benefits from rTMS and that the treatment was well tolerated, it was agreed to offer the patient a maintenance rTMS program that comprised weekly rTMS sessions. Maintenance rTMS was initiated at 11 weeks following the initial rTMS treatment phase, with resting motor threshold again identified as 31% of maximal stimulator output, and an identical stimulation protocol delivered. Ten weekly rTMS sessions were delivered over an 11-week period (one session missed due to patient availability). The patient verbally reported that the maintenance treatments were helping, and this was confirmed with the BDI (score 10) and PHQ-9 (score 4) that indicated depressive symptoms had reduced to a minimal/nil level. Given this progress, the patient agreed to stop treatments. No additional adverse events were noted beyond the previously reported transient hearing and concentration symptoms identified following prior rTMS treatment. At 3 months following the maintenance phase, the research team contacted the patient to ascertain whether clinical gains had been maintained. The patient reported minimal-mild symptoms of the BDI (score 11) and PHQ-9 (score 8), with scores still indicative of a clinically meaningful improvement from Baseline.

## Discussion

To our knowledge, this is the first report of an extended rTMS treatment protocol, comprising acute daily rTMS treatments and subsequent weekly maintenance sessions with long-term follow-up data, in a chronic stroke patient with a lesion of the left frontal cortex. Findings from this case suggest high-frequency rTMS of the left DLPFC appears beneficial for reducing symptoms of depression in a patient with structural damage of the left frontal cortex. Improvements in depressive symptoms were clinically meaningful after the intensive acute daily treatment phase. Improvements appeared to persist for ~10 weeks before noticeable reduction by the patient and the PHQ-9 no longer indicating a clinically meaningful improvement from baseline. Delivery of maintenance sessions appeared effective, preventing further decline, with benefits noted out to 3 months after cessation. While rTMS delivered in close proximity to the lesion could impose an increased risk of seizure, our findings provide early indication that treatment provided to a patient with chronic stroke, screened for rTMS safety, and without history of seizure, was safe. The treatment program appeared well tolerated by the patient, suggesting rTMS may be a promising therapy for ongoing management of post-stroke depression.

Although previous literature suggests structural and functional properties of the anatomical rTMS target can attenuate responses (25, 26), this case appears to indicate that clinically meaningful improvements can be achieved despite a lesion of the frontal cortex. Growing evidence suggests that the efficacy of rTMS for depression is associated with functional connectivity between DLPFC and the subgenual cingulate cortex (38, 39). The subgenual cingulate cortex plays a role in regulating emotion, and in this case, appears to be spared by the lesion. It may be that rTMS pulses stimulated a region where a small, viable part of the cortex remained intact with residual connectivity with the subgenual cingulate cortex. Further studies evaluating functional connectivity between DLPFC and the subgenual cingulate cortex in patients with lesions of the frontal cortex might help elucidate this mechanism.

The improvements observed in this case study following 10 daily sessions of rTMS are similar to those reported in previous post-stroke depression trials (23–25). Our findings provide new clinical insight to suggest that rTMS may be a beneficial treatment for longer-term management of depression after stroke. Clinicians should monitor depressive symptoms following rTMS treatment and identify patients likely to benefit from ongoing management with maintenance. Although regularity of rTMS sessions during maintenance phases vary in the literature (40), our early observation is that weekly sessions prevent further decline.

Finally, while this case is unique in the literature, providing insight as to whether rTMS of the left DLPFC is effective in treating depression for a patient with a left frontal lesion and suggesting that maintenance treatments can lead to more persistent benefits, we suggest caution when interpreting these results. Most notably, this is a case study, and subsequent appropriately powered trials are now required. Furthermore, it is unclear to what extent the stimulation directly overlapped with the lesion as the rTMS coil was positioned by anatomical landmarks. To further explore the relationship between stimulation target and the lesion, future studies should employ neuronavigation. Finally, we acknowledge this study did not include a sham treatment to validate effectiveness of rTMS for depression or decipher any placebo effect. However, we note early evidence from randomized trials in post-stroke depression provides some support for rTMS efficacy (23–25). Despite these limitations, this case study provides novel insight to longer term treatment and outcomes of rTMS for post-stroke depression that might prove valuable for clinical management or informing future trials.

## Data Availability Statement

The raw data supporting the conclusions of this article will be made available by the authors, without undue reservation.

## Ethics Statement

This study involving a human participant was reviewed and approved by the University of South Australia Human Research Ethics Committee. The patient provided their written informed consent to participate in this study.

## Author Contributions

BH, AC, and SH contributed to study design. BH, AC, LG, and SH contributed to analysis, interpretation, and drafting of the manuscript. All authors contributed to the article and approved the submitted version.

## Funding

The project was supported by a grant from the Breakthrough Mental Health Foundation. BH was supported by an NHMRC fellowship (GNT1125054).

## Conflict of Interest

BH holds a paid consultancy role for Recovery VR and has a clinical partnership with Fourier Intelligence. The remaining authors declare that the research was conducted in the absence of any commercial or financial relationships that could be construed as a potential conflict of interest.

## Publisher's Note

All claims expressed in this article are solely those of the authors and do not necessarily represent those of their affiliated organizations, or those of the publisher, the editors and the reviewers. Any product that may be evaluated in this article, or claim that may be made by its manufacturer, is not guaranteed or endorsed by the publisher.
